# The Figure Memory Test: diagnosis of memory impairment in populations with heterogeneous educational background

**DOI:** 10.1590/1980-57642021dn15-020004

**Published:** 2021

**Authors:** Ricardo Nitrini, Sonia Maria Dozzi Bucki, Mônica Sanches Yassuda, Helenice Charchat Fichman, Paulo Caramelli

**Affiliations:** 1 Department of Neurology, Faculdade de Medicina, Universidade de São Paulo ‒ São Paulo, SP, Brazil; 2 Departament of Neurology, Hospital Santa Marcelina ‒ São Paulo, SP, Brazil; 3 Gerontology, School of Arts, Sciences and Humanities, Universidade de São Paulo ‒ São Paulo, SP, Brazil.; 4 Department of Psychology, Pontifícia Universidade Católica do Rio de Janeiro ‒ Rio de Janeiro, RJ, Brazil.; 5 Behavioral and Cognitive Neurology Group, Faculdade de Medicina, Universidade Federal de Minas Gerais ‒ Belo Horizonte, MG, Brazil.

**Keywords:** dementia, mild cognitive impairment, Alzheimer disease, memory, education, brief cognitive screening battery, demência, comprometimento cognitivo leve, doença de Alzheimer, memória, educação, bateria breve de rastreio cognitivo

## Abstract

Diagnosis of cognitive impairment is usually difficult in low-educated individuals. The Brief Cognitive Screening Battery (BCSB) was designed as a screening tool for the evaluation of cognitive impairment in low-educated individuals, but it may also be used for evaluating individuals with high educational level.

**Objectives:**

To perform a narrative review analyzing the origin of the BCSB, to report all studies that have used the Figure Memory Test (FMT) of the BCSB, and to demonstrate that it is a useful battery for regions where populations have heterogeneous educational background.

**Methods:**

We performed a search in PubMed, SciELO, and LILACS using the terms “Brief Cognitive Screening Battery” and “Brief Cognitive Battery”.

**Results:**

We obtained 49 papers from PubMed, 32 from SciELO, and 28 from LILACS. After the exclusion of duplicate papers, 54 publications were obtained; five more studies were included from previous knowledge of the authors. Twenty-four papers were related to the impact of education on performance, diagnostic accuracy, cutoff scores and normative studies. The delayed recall of the FMT showed the best accuracy for the diagnosis of dementia with a cutoff score of ≤5 in different education levels. In 35 papers, the FMT of the BCSB was used in clinical studies with different settings, from outpatient memory clinics to epidemiological studies and evaluation of Amazon river basin dwelling individuals, and it was always considered to be easy to apply.

**Conclusions:**

The FMT of the BCSB is an easy and short tool for the diagnosis of dementia in populations with heterogeneous educational background.

## RESUMO.

O diagnóstico de comprometimento cognitivo costuma ser difícil em indivíduos de baixa escolaridade. A Bateria Breve de Rastreio Cognitivo (BBRC) foi elaborada como uma ferramenta de triagem para avaliação do comprometimento cognitivo em indivíduos de baixa escolaridade, mas também pode ser utilizada para avaliar indivíduos com alto nível educacional.

**Objetivos:**

Realizar uma revisão narrativa da origem da BBRC, relatar todos os estudos que utilizaram o Teste de Memória de Figuras (TMF) da BBRC e demonstrar que essa é uma bateria útil para regiões cuja população possui formação educacional heterogênea.

**Métodos:**

Foi realizada uma busca no PubMed, SciELO e LILACS utilizando os termos “*Brief Cognitive Screening Battery*” e “*Brief Cognitive Battery*”.

**Resultados:**

Um total de 49 artigos no PubMed, 32 na SciELO e 28 na LILACS foram obtidos. Após a exclusão de artigos duplicados, totalizaram-se 54 publicações; mais cinco estudos foram incluídos com base no conhecimento prévio dos autores. Vinte e quatro artigos foram relacionados ao impacto da educação no desempenho, à precisão, pontuações de corte e estudos normativos. A recordação diferida do TMF mostrou a melhor precisão para o diagnóstico de demência com uma pontuação de corte de ≤5 em diferentes níveis educacionais. Em 37 artigos, o TMF da BBRC foi utilizado em estudos clínicos em diferentes ambientes, de ambulatórios de clínicas especializadas a estudos epidemiológicos e na avaliação de indivíduos residentes nas margens de rios da bacia amazônica, e sempre foi considerado de fácil aplicação.

**Conclusões:**

O TMF da BBRC é uma ferramenta simples e rápida para o diagnóstico de demência em população com heterogeneidade educacional.

## INTRODUCTION

In 1990, we started a project whose objective was to investigate whether dementia caused by neurodegenerative diseases, in particular Alzheimer disease, was more frequent among people with higher socioeconomic level, while vascular dementia and dementia of other non-degenerative etiologies were more frequent in individuals of lower socioeconomic status. To carry out the research, a specialized outpatient clinic was created at the Hospital das Clínicas of the School of Medicine of Universidade de São Paulo, in São Paulo, Brazil, a large public university hospital. Cases seen at a private clinic were also included in order to improve the sample regarding higher socioeconomic levels.

In the designing phase of the project, it was clear that it would be difficult to establish the diagnosis of dementia in individuals with low education, especially illiterates. Tests used in other countries would probably be insufficient or could lead to misdiagnosis. The Mini-Mental State Examination (MMSE) had proved to be very inadequate in previous studies carried out in low- and middle-income countries, such as in China, many years before, when Katzman tried to use the MMSE for the diagnosis of dementia.^1^

One of the authors of the present study (RN) had already carried out a study at *Hospital das Clínicas*, of the School of Medicine of Universidade de São Paulo, using a version of the MMSE in a sample of 362 individuals without cognitive impairment, including 23 illiterate and 69 with 1 to 3 years of schooling. Scores ranged from 17 to 27 among illiterates, and from 18 to 30 among those with schooling from 1 to 3 years.^2^ In another study carried out in Brazil, it was found that the most suitable MMSE cut-off score for dementia diagnosis among illiterates was 13 (5^th^ percentile value), posteriorly published.^3^ It was clear that the use of the MMSE, particularly if considering the cut-off score originally proposed by the authors (24 points),^4^ would not be adequate to screen for dementia among patients with no or low education.

A few months before the study started, Morris et al.^5^ published the Consortium to Establish a Registry for Alzheimer's Disease (CERAD) battery for the clinical and neuropathological diagnosis of Alzheimer disease (AD). Although it would be an interesting set of tests to be used, the memory test (word list) would still require reading, thus preventing its use among illiterates.

Faced with this difficulty and not knowing any method that could be used for adequate cognitive assessment of illiterates and individuals with low schooling, especially memory, we developed the Brief Cognitive Screening Battery (BCSB), which includes the Figure Memory Test (FMT).^6^ http://www.demneuropsy.com.br/imageBank/suplementar/Brief_Cognitive_Screening_Battery.pdf

This test consists of presenting a sheet of paper with 10 simple black and white drawings. The figures are named by the subjects (Naming score), who are then asked to recall each drawing immediately, without having been told that the figures should be memorized (Incidental Memory score). Subsequently, the figures are displayed again and the subjects are asked to memorize them for 30 seconds, after which they should be recalled (Immediate Memory score). The same procedure is then repeated to obtain the Learning score (or encoding). Then, after the Verbal Fluency test (animals in 1 minute) and the Clock Drawing Test (CDT), which are interference tests, subjects are asked to recall the figures shown previously (Delayed Recall score). Finally, a sheet of paper with the 10 target figures, mixed with 10 new distracting figures, is presented and the subjects are asked to recognize those figures displayed originally (Recognition score). Scoring on these subtests ranges from 0 to 10 points (except for the Verbal Fluency test).^6^ ^,^ ^7^

The study continued with the inclusion of 100 patients with dementia and the conclusion was that the socioeconomic level had no influence on the relative frequency of neurodegenerative, vascular, and other dementia subtypes.^8^ When a sub sample was paired by age and education with controls, it was possible to verify that the delayed recall of the FMT (DR-FMT) showed excellent sensitivity (93.3%) and specificity (96.6%) in differentiating between patients with mild to moderate dementia from controls, with a cut-off score of ≤5. The delayed recall also obtained the largest area under the ROC curve (AUROC), when compared to the other BCSB subtests.^6^ Since then, this test started being applied as a screening tool for the diagnosis of dementia and memory impairment in the clinical setting and in several other studies.

The aim of this review was to include all studies that provided evidence for their identification as a relevant cognitive test or their psychometric properties, since inception. Another objective was to report all studies that have used the BCSB or the FMT of the BCSB to either confirm memory impairment or exclude it. Finally, the most important objective was to disseminate the FMT of the BCSB, which is free of charge, so that it may be used for dementia diagnosis in populations with heterogeneous educational background.

## METHODS

A search was performed in PubMed, SciELO (Scientific Eletronic Library Online), and LILACS (Latin American and Caribbean Health Sciences Literature) using the terms “Brief Cognitive Screening Battery” and “Brief Cognitive Battery”. Studies that the authors were aware of, but had not been found in the bibliographic search, were also included.

All papers in which the BCSB was used were included. Papers where the term “brief cognitive battery” was used for other cognitive tests were excluded. All abstracts and relevant papers were fully read by the authors. The selected studies were presented in chronological order, according to their year of publication and the types of papers, which were classified into two types. The former demonstrated low or no impact of education or were related to construct validity or to normative studies. The latter were represented by papers in which the BCSB, particularly the DR-FMT, was used either to confirm memory impairment or to exclude it. Manuscripts in English, Spanish, and Portuguese were included.

## RESULTS

The search in PubMed identified 49 papers in SciELO 32 and 28 in LILACS. After the exclusion of duplicate papers, 54 publications were obtained. Five additional papers were included from previous knowledge of the authors. Therefore, the final number of selected articles was 59.

### Influence of Education, Construct validity, and normative studies

In the epidemiological studies of dementia conducted in Catanduva, Brazil, the BCSB was applied in the prevalence study^9^ and in the incidence study conducted 3.5 years later.^10^ Data obtained in the prevalence study allowed comparing the performance of 51 cognitively unimpaired individuals, 23 being illiterate, in dementia screening tests.^7^ ^,^ ^9^ The scores of the illiterate group were significantly lower in the MMSE, Boston Naming Test (15-item version), in the CERAD Word List memory test (immediate and delayed recall), CDT, and Verbal Fluency (animals in 1 minute), but they did not differ from those of the literate individuals in the immediate and delayed recall of the FMT from the BCSB. FMT scores did not differ when the illiterate group was compared to the whole literate one, neither when the literate group was divided according to mean years of schooling. The means of three trials, both for the CERAD and the BCSB, were taken into account for the immediate recall scores. This was the first demonstration of the non-significant or absent impact of schooling on the performance on the FMT of the BCSB.^7^

In 2006, Takada et al. compared the accuracy of two memory tests, the delayed recall from the CERAD Word List test and the DR-FMT of the BCSB in diagnosing dementia. In the study sample, extracted from the Catanduva incidence study,^10^ there were 17 illiterate and 17 non-illiterate subjects with dementia and 23 illiterate and 28 non-illiterate ones without it. The DR-FMT showed higher accuracy than the delayed recall test of the CERAD among illiterates, whereas both tests had equivalent accuracy among literate individuals. However, there was a trend for higher accuracy for the DR-FMT when the entire sample was analyzed. The main conclusion of that study was that the DR-FMT of the BCSB could represent “an alternative for the diagnosis of dementia in populations with high proportion of illiterates.”^11^

In 2007, Christofoletti et al. investigated the influence of schooling on the performance in the BCSB by evaluating 176 older adults, 60 of them with cognitive decline. The authors concluded that education did not have a significant influence on the FMT and that this aspect favors dementia diagnosis in populations characterized by illiteracy or low education. The authors suggested that there is no need of adjustment of the cutoff scores for the FMT according to education and that the BCSB consists of several simple procedures for cognitive screening and monitoring for any potential decline.^10^ The authors reported a significant influence of schooling for Verbal Fluency and CDT, and indicated that these BCSB interference tests may need education-adjusted cut-off scores.^12^

The FMT was originally developed for individuals with low educational level, but in epidemiological studies and in the clinical scenario it was possible to observe that it was also useful for the diagnosis of memory impairment in individuals with higher educational levels. However, it is was only in 2007 that a study effectively confirmed this hypothesis.^13^ In a sample of 73 patients with mild AD dementia and 94 controls with middle and higher levels of education, the accuracy of the BCSB in the diagnosis of dementia due to AD was investigated. There was also the objective of developing a mathematical model that included the most discriminating tests of the BCSB. The DR-FMT had the highest AUROC (0.931) when compared to the other tests of the BCSB, with sensitivity of 82.2% and specificity of 90.4% with a cut-off of ≤5. A mathematical model that included three subtests, namely delayed recall and learning of the FMT, and Verbal Fluency (animals in one minute), obtained a high accuracy, with an AUROC of 0.917.^13^

In another study from our group, brief cognitive tests were used to evaluate 105 patients without cognitive complaints seen at a public general neurology outpatient clinic. Education-adjusted cut-off scores were used for the MMSE (<18 for illiterates; <21 for 1‒4 years, <24 for 5‒8 years, and <26 for more than 8 year); for semantic Verbal Fluency (<9 for illiterates, <12 for 1‒7 years, and <13 for ≥8 years). Tests that had the same cut-off for all education levels were the Digit Span with <5 and <3 for the Forward and Backward, retrospectively; CDT <9 and DR-FMT <7. Performance below these cut-off scores were observed in the MMSE (20.0%), Digit Span Forward (29.5%), Digit Span Backward (20.9%), Verbal Fluency (27.6%), CDT (40.0%), and DR-FMT (14.2%). The DR-FMT showed the lowest percentage of false-positives in these subjects without cognitive complaints than the other screening tests used in that study.^14^ This finding suggests that the DR-FMT is less biased by sociodemographic variables, and that may be one the reasons for presenting higher specificity for the diagnosis of cognitive impairment.^14^

Brief language tests were investigated for the detection of individuals with cognitive decline in a sample of 74 individuals classified into the Clinical Dementia Rating (CDR) - CDR 0 (n=33), CDR 0.5 (n=17), and CDR 1.0 (n=24). The tests used were the Naming subtest of the BCSB, CERAD version of the Boston Naming Test (15 items), Verbal Fluency (animals from the BCSB and fruits) and the language items of the MMSE. Although there were significant differences between control subjects (CDR 0) and CDR ≥0.5 in all tests, the CDR 0.5 group performed better than the CDR 1 group only in animal fluency. Stepwise multiple regression revealed that fruit fluency, animal fluency, and Naming of the BCSB were the best discriminators between patients (CDR 0.5 and 1) and controls (CDR 0). These tests were less influenced by schooling, when literate and illiterate individuals were compared.^15^

In a study to investigate the cognitive performance of healthy elderly with and without subjective memory complaints, there was no difference between the performance of the groups of complainers and non-complainers in the different cognitive tests of the BCSB.^16^

In 2008, the influence of age, gender, and educational level on the performance in the BCSB was investigated in a sample of 325 individuals (207 women), with a mean age of 47.1 (±16.8) years, ranging from 19 to 81, and a mean of 9.8 (±5.0) schooling-years. There was a significant negative correlation between age and performance in the DR-FMT (-0.339) and a significant positive correlation between years of schooling and performance in the DR-FMT (0.231). Although these correlations were significant, they were low in magnitude, especially for the DR-FMT and education.^17^

Another study on subjective memory impairment (SMI) was carried-out in the Amazon rainforest. In a sample composed of 163 subjects (82 women) with a mean age of 62.3 years (50‒94 years), 110 of whom were illiterate, a very high prevalence of SMI (70%) was observed, being more frequent among women. Age and education did not impact SMI. Subjects with SMI had significantly more somatic and psychiatric symptoms, as well as lower scores on the MMSE, but not on the DR-FMT. It was also very important to observe that the FMT was very friendly and easy to apply to this population, which was able to obtain a median of 10 right answers in Naming amidst 10 figures.^18^

In the BCSB, the CDT is an interference test, yet it is also useful for the diagnosis of cognitive impairment in individuals with middle and high education. When used in individuals with low education (less than four years), it takes more time to be concluded and it is less informative, as these individuals may have difficulty to complete paper-and-pencil visuo-spatial constructive tasks. Castro et al. analyzed the effect of excluding the CDT from the BCSB on the delayed recall of the FMT.^19^ The mean time to complete the BCSB, which is usually about 8 minutes,^13^ was reduced by 2 minutes and 37 seconds and the DR-FMT kept its high accuracy in diagnosing dementia. However, there was a significant difference between the delayed recall of the two versions of the test, the values being higher without the CDT, most likely, due to the fact that the interval between Learning and Delayed Recall was shortened.^19^

Charchat-Fichman et al. analyzed the performance of healthy older adults on the Rey Auditory Verbal Learning Test (RAVLT), describing the effects of age, gender, and education. To investigate its construct validity, the RAVLT was compared with the delayed recall and recognition scores of the FMT of the BCSB. A significant correlation was found between the delayed recall of the RAVLT and the DR-FMT (r=0.5, p<0.01).^20^

Wachholz and Yassuda evaluated the relationship between the interpretation of proverbs, education, executive functions, and episodic memory in 67 older adults. DR-FMT was used to examine episodic memory. The ability to correctly interpret proverbs was strongly associated with education and executive functions, although there was a weaker relationship with episodic memory.^21^

In 2012, the CERAD Battery and the BCSB were compared in the differential diagnosis of healthy controls, patients with major depression and patients with AD. The most relevant tests for this differentiation were the episodic memory tests of both batteries (Word List and DR-FMT, respectively). The delayed recalls (without cues) of both tests were able to differentiate cognitive decline associated with depression from AD. DR-FMT recognition was superior to the Word List for this differentiation.^22^

In 2014, Brucki and Nitrini published a more extensive report of their findings to evaluate the prevalence of subjective memory impairment, cognitive impairment no dementia (CIND), and dementia in a population living in the Amazon river basin. The DR-FMT and semantic verbal fluency from the BCSB were used for diagnostic purposes, together with the MMSE and a range of tests adapted to the local cultural context. Among older adults (65+ years), there was a 12.3% prevalence of dementia and 7.7% of CIND.^23^

In 2016, Fichman-Charchat et al. evaluated the use of the BCSB for the diagnosis of mild AD dementia in a geriatric outpatient unit of a public hospital. Patients with AD were compared to a non-AD group. The DR-FMT was the best diagnostic parameter with sensitivity and specificity superior to 80%, although the cutoff score was lower in this study (<5). There are several reason for the low cutoff score in this sample: mean age (79.57±6.98 years), high prevalence of depression (29.5%), previous vascular disease (3.3%), systemic arterial hypertension (79.5%), high level of cholesterol (23%), and diabetes mellitus (17.2%). Besides, the inclusion of patients with MCI among the non-AD may have influenced the sensitivity, specificity, and cutoff of the DR-FMT of the BCSB in this sample.^24^

An epidemiological study on late-life depression (LLD) was carried out by investigating 639 community-dwelling individuals aged 75+ years and with low educational level in Caeté, Minas Gerais, Brazil. After excluding 174 individuals with dementia, 465 individuals were investigated for depression. LLD was diagnosed in 54 (11.6%) individuals and their cognitive and functional performances were evaluated with the MMSE, the BCSB, and the Pfeffer's Functional Activities Questionnaire (FAQ). A very important finding of this study was that the DR-FMT scores of 411 individuals without depression and 54 with depression were not different and for both groups the median score was 7 with the 25^th^ and 75^th^ percentiles being 6 and 8.^25^

In 2017, Lima et al. evaluated the performance on basic cognitive tasks, instrumental activities of daily living, and depressive symptoms of a community-based sample of 264 aged adults in Rio de Janeiro (Brazil). The BCSB, Lawton’s, and Pfeffer’s activities of daily living indexes, and the Geriatric Depressive Scale (GDS) were employed. One relevant finding was that the scores in the DR-FMT did not differ between education levels 1‒4 and 5‒8 years of schooling. However, when the performance of individuals in these education levels was compared to that of individuals with ≥10 years of schooling, there was a modest but significant difference (p<0.05).^26^

In 2019, a study compared the MMSE with the BCSB as screening tests for dementia in low-educated people. A sample of 112 individuals was examined by two evaluators, who were blinded to each other’s results. Education level influenced the results of the MMSE and CDT, but not of the DR-FMT. The Verbal Fluency test was influenced only by higher educational levels. It took three minutes longer to apply the BCSB than to apply the MMSE.^27^

The Recommendations of the Brazilian Academy of Neurology for the cognitive, functional, and behavioral assessment of Alzheimer disease was published in 2011 by Chaves et al.^28^ The BCSB was included among the tests to be employed in Brazil, as the FMT and semantic Verbal Fluency met the minimum validation requirements, having significant normative data and wide application in the Brazilian population. In that consensus meeting, it was commented that in spite of the wide application of the BCSB in Brazil, normative data for different age and education individuals were still needed.^28^

Although the BCSB had been used for more than 20 years, there was not a complete study on normative data stratified by age and education up to 2017, when Yassuda et al. published data from 240 community-dwelling older adults from a sub-district in the city of São Paulo.^29^ Inclusion criteria were scored above the education-adjusted cutoff points in the MMSE and below six points on the Geriatric Depression Scale GDS. The results of the DR-FMT according to age and education are reproduced in Tables 1 and 2).

**Table 1.** Delayed recall of the Figure Memory Test of the Brief Cognitive Screening Battery according to age.

Age	65‒74 years	(75 years	p-value
Delayed Recall			<0.001
Median	8	8	
Mean (SD)	8.22 (1.30)	7.55 (1.06)	
Minimum	4	4	
Maximum	10	10	

Source: data from Yassuda et al.^29^

SD: standard deviation.

**Table 2.** Delayed recall of the Figure Memory Test of the Brief Cognitive Screening Battery according to education

Education (years)	0 (illiterate)	1‒4	(5	p-value
Delayed recall				0.668
Median	8	8	8	
Mean (SD)	7.80 (1.68)	8.07 (1.18)	8.20 (1.24)	
Minimum	4	5	6	
Maximum	10	10	10	

Source: data from Yassuda et al.^29^

SD: standard deviation.

The results from that study confirmed that the DR-FMT was not heavily influenced by education and that it can be used to identify memory impairment in older adults with different educational backgrounds. The results also confirmed that the semantic Verbal Fluency test and the CDT were influenced by age and education (data not shown here).

In 2018, Araujo et al. evaluated the impact of age and schooling on the performance of the BCSB of 470 community-dwelling aged adults in Rio de Janeiro (Brazil). The mean age was 72.77±7.06 years, with mean schooling of 9.54±5.32 years. The ANOVA showed that individuals aged older than 80 years presented worse performance in memory tasks compared with younger age groups (60‒69 or 70‒79 years). Pearson’s correlation analysis indicated that FMT was negatively correlated with age. Regarding education, there was no difference between groups in FMT, only in executive and global functioning tasks.^30^ The present results indicated a dissociation, in which age affected memory function measured by FMT, and schooling affected executive, language, and global cognitive functions measured by Verbal Fluency, CDT, and MMSE. The present results indicated that BCSB is a good tool for cognitive screening that was not influenced by interactions between age and schooling. Borges et al. studied a subsample of 264 aged adults in this community, finding a strong correlation between FMT and instrumental activity of daily living. Table 3 shows the performance in FMT stratified by education.^31^

**Table 3.** Figure Memory Test according to attained years of education.

Education (years)	0 Mean (SD)	1‒4 Mean (SD)	5‒8 Mean (SD)	9‒11 Mean (SD)	(12 Mean (SD)
Naming	9.65 (0.70)	9.67 (0.89)	9.86 (0.59)	9.84 (0.74)	9.92 (0.45)
Incidental Memory	5.76 (1.48)	5.48 (1.59)	5.63 (1.28)	5.41 (1.41)	5.46 (1.51)
Immediate Memory	7.59 (1.50)	7.66 (1.56)	7.76 (1.44)	7.87 (1.45)	7.85 (1.49)
Learning	8.53 (0.94)	8.27 (1.58)	8.41 (1.22)	8.73 (1.33)	8.62 (1.39)
Delayed recall	7.71 (1.61)	7.03 (2.42)	7.47 (1.88)	7.45 (2.16)	7.73 (2.02)
Recognition	9.94 (0.24)	9.68 (0.94)	9.76 (0.61)	9.80 (0.53)	9.79 (0.67)

Source: data from Araújo et al.^30^

SD: standard deviation.

These studies demonstrate that the scores in DR-FMT of the BCSB suffer little or no effect from schooling. It was also possible to verify from studies comparing cases and controls, that the best cutoff score for the diagnosis of dementia was ≤5. Based on the normative values (Tables 2 and 3), if we use 2 standard deviations (SD) below the mean, the best cutoff score of the DR-FMT for the diagnosis of dementia would also be ≤5. For mild cognitive impairment, score ≤6 (1.5 SD below the mean) may be used.

### Clinical studies including the BCSB FMT as a test for memory impairment

Christofoletti et al. analyzed the effects of two interventions on cognition and balance of 54 institutionalized elderly patients with mixed dementia. Patients were divided into 3 groups: physiotherapy, occupational therapy, and physical education for group 1, only physiotherapy for group 2, and group 3 was the control. Cognitive functions were analyzed with the MMSE and BCSB. Univariate analysis indicated some benefits of the interdisciplinary intervention on semantic verbal fluency and CDT of the BCSB.^32^

In a study aimed to compare the effects of pain and opioids in the cognitive functions of patients with cancer, screening tests were used, including the BCSB. It was possible to find that pain severity correlated negatively with scores in several cognitive tests (MMSE and items of the BCSB) whereas opioids did not correlate with any measures of cognitive performance.^33^

To evaluate the relationship between cortisol levels and memory performance among healthy older adults (n=40), and those diagnosed with mild cognitive impairment (n=39) and AD (n=40), Souza-Talarico et al. used the FMT.^34^ Higher cortisol levels were associated with better memory performance in healthy older adults, while higher cortisol levels were correlated with poorer memory performance in MCI subjects. No correlation between cortisol levels and memory was found among individuals with AD dementia. Therefore, the relationship between cortisol levels and memory performance may vary according to the presence or absence of cognitive impairment.

In 2010, Matioli and Caramelli investigated the diagnostic value of brief cognitive tests in differentiating vascular dementia (VaD) from AD, using the MMSE, BCSB, EXIT 25 battery and the copy of the clock drawing (CLOX 2), and category and letter fluency tests. VaD patients performed worse than AD patients in category fluency, letter fluency, and CLOX 2, while AD cases performed worse than VaD patients in the DR- FMT. The value of such instruments in differentiating VaD from AD proved to be very limited.^35^

Vianna and Yassuda applied the BCSB in an investigation on the relationship between memory complaints, education, cognitive performance, and symptoms of depression and anxiety among 67 older adults. Anxiety symptoms were associated with memory complaints, which were not associated either with cognitive performance or depressive symptoms.^36^

Lima-Silva et al. investigated the effects of cognitive training based on metamemory and mental images in individuals with 3 to 15 years of schooling. An intervention group (n=37) participated in five training sessions while 32 members of the control group were submitted to the tests only, pre- and post-training sessions. The DR-FMT was pre-selected as the test to evaluate the results of the intervention and it was able to detect significant improvement after cognitive training.^37^

Pereira et al. performed a longitudinal study in community-dwelling older adults without dementia to evaluate cognitive decline over the years. In 2006, 168 individuals were evaluated for the first time, and, in 2009, 73 of these subjects were reevaluated in relation to cognition and functionality through the MMSE, BCSB, and FAQ. After three years, there was a discrete, yet significant decline in the MMSE, FAQ, and Verbal Fluency. There was no significant decline in the DR-FMT.^38^

In 2011, Aramaki and Yassuda performed a follow-up evaluation after 18 months of 37 aged individuals who had participated in five sessions of cognitive training based on metamemory and mental images.21,24 The objectives were to detect possible maintenance of gains after first post-training assessment and to evaluate the impact of a training booster session. The BCSB was used together with other tests as outcome variables. Results indicated that the score of DR-FMT improved after the booster session, which suggests that reviewing training content periodically may help maintain cognition in adulthood and old age.^39^

In 2011, Bawden et al. investigated the cognitive performance of 17 patients with diagnosis of obstructive sleep apnea in comparison to 20 age- and education-matched healthy controls. The testing battery included the MMSE, BCSB, Digit-Symbol, and phonemic verbal fluency tests. Patients performed significantly worse than controls in most tests, including in the DR-FMT from the BCSB.^40^

In a study to investigate weight training effects on cognitive functions, 34 patients with AD were allocated into two groups: Training Group (TG) and Social Gathering Group. MMSE and the BCSB were used. After 16 weeks of training, there was no evidence of improvement in cognitive functions.^41^

In 2012, Yassuda et al. assessed the relationship between frailty and cognitive performance among 384 community-dwelling older adults in a poor sub-district of the city of São Paulo. The BCSB and the MMSE were the tests used. Frail older adults had significantly lower scores in all tests, concluding that interventions should consider that these forms of vulnerability may occur simultaneously.^42^

Another important study that included the BCSB and the MMSE was the investigation of determinants of the cognitive performance of older adults living in an impoverished sub-district of São Paulo. Age, gender, schooling, depressive symptoms, and systolic blood pressure level had a significant impact on the MMSE; age and education were predictors of Verbal Fluency; and age and GDS (but not education) predicted the DR-FMT score.^43^

In 2013, Frota et al. evaluated the cognitive impairment of patients with Wilson disease (WD) by correlating the cognitive findings with changes in magnetic resonance imaging (MRI). Patients with WD performed significantly worse than controls in several tests, including MMSE, Dementia Rating Scale, phonemic verbal fluency (FAS), verb generation, Digit Span Forward, Stroop test, Frontal Assessment Battery, and the DR-FMT of the BCSB. There was also a good correlation between cognitive impairment and an MRI scale.^44^

Porto et al. used screening tests to test whether it would be possible to differentiate individuals diagnosed with MCI from those who had subjective memory impairment without dementia. Two delayed recall tests were added to the MMSE. The association of the MMSE with the DR-FMT and a phonemic verbal fluency (letter P) was able to differentiate mild cognitive impairment (MCI) from subjective memory impairment without dementia.^45^

In a study on memory complaints, 301 community-dwelling older adults completed the Memory Complaint Questionnaire, GDS, and cognitive tests, including the BCSB. Memory complaints were not associated with low cognitive performance, but were correlated with female gender, low education, and depressive symptoms.^46^

The BCSB and the MMSE were applied to assess the cognitive status of a group of patients with stroke sequelae who were submitted to two different physiotherapeutic methods.^47^

To evaluate the association between performance prediction, an aspect of metamemory, and objective memory performance, 359 community-dwelling older adults completed the BCSB and answered a simple question. The question was: “If someone showed you a sheet with the drawings of 10 pictures to observe for 30 seconds, how many pictures do you think you could remember without seeing the sheet?”. Regression analyses showed that sociodemographic variables did not influence memory prediction, which was modestly associated with the Immediate Memory subtest of the BCSB FMT.^48^

To investigate the neuropsychological profiles of patients with MCI from a geriatric clinic of a tertiary public hospital in Rio de Janeiro, Brazil. The MMSE, BCSB, and Lawton and Katz Daily Living Activities were used to identify and map the neuropsychological profile. The most frequent type of MCI was the non-amnesic dysexecutive MCI.^49^

In a study to investigate the association between advanced activities of daily living (AADL) and cognitive performance, 302 community-dwelling subjects without cognitive impairment were evaluated. The MMSE and the BCSB were used for assessing cognitive performance and the GDS was also employed. When the subjects were divided into very active or little active in AADL, there was no association between higher AADL and better cognitive performance. Depressive symptoms and sociodemographic factors were probably better modulators of AADL and cognitive performance.^50^

Christofoletti et al. investigated the influence of cognitive processes on the gait of patients with Parkinson disease (PD) and AD in dual task. The BCSB was used for this study and it was possible to verify that executive functions (assessed by the CDT) have the most relevant cognitive influence in complex tasks.^51^

A study investigating 113 individuals with PD (78 men), with mean age of 68.1±9.4 years, mean formal education of 3.9±3.2 years and duration of symptoms, that is, mean length of time since the patients developed PD of 7.7±5.2 years, were screened for dementia with the BCSB. Dementia was detected using the Mattis Dementia Rating Scale in 46 patients (40.7%). All subtests of the FMT, except Naming, were able to identify patients in the screening phase.^52^

In a study of prevalence of dementia in Tremembé, São Paulo State, Brazil, the examination for the diagnosis of dementia was performed in a single phase. The DR-FMT, with a cutoff score of ≤5, was included as a criterion for the selection of patients, together with the MMSE and the FAQ, to participate in a consensus meeting for diagnosis of dementia.40 Results indicated prevalence rates of 17.5% for dementia and 19.5% CIND. These prevalence rates were influenced by age and educational level. Results indicated prevalence rates of 17.5% for dementia and 19.5% CIND. These prevalence rates were influenced by age and educational level.^53^

Decision-making in cognitively unimpaired individuals may be influenced by education. To evaluate this hypothesis, a sample of illiterate and low educated individuals completed the Iowa Gambling Test. To be included in the study, individuals should have normal performance in several cognitive tests and questionnaires, including the BCSB, MMSE, Digit Span Forward and Backward, Raven's Colored Progressive Matrices, Wisconsin Card Sorting Test, GDS, and Geriatric Anxiety Inventory. The performance in the Iowa Gambling Test was influenced by education, with worse scores among the illiterate.^54^

Leite et al. investigated the performance of illiterate and low-educated older adults on different versions of the Boston Naming Test (BNT). The BCSB was used for excluding individuals with cognitive impairment. The main conclusion was that the Brazilian adapted version of the BNT was more appropriate for the low-educated population.^55^

To investigate the frequency of cytokine single nucleotide polymorphisms (SNP) in a sample of healthy, CIND and dementia subjects, the MMSE, BCSB, and a functional questionnaire were used for diagnosis. The study suggested a potential role for certain cytokine SNP in the development of CIND and dementia.^56^

The integrity of white matter structure was investigated with MRI with diffusion tensor imaging (DTI) acquisition in a sample of 31 aged subjects (23 cognitively healthy and 8 with CIND), with 2.2±1.9 years of education. One of the main objectives was to investigate correlations between the BCSB and the RAVLT performance with DTI parameters. The DR-FMT correlated with frontotemporoparietal connection bundles, with the hippocampal part of the cingulum bundle bilaterally and with the right superior longitudinal fasciculus. The RAVLT learning and delayed recall scores also correlated with the hippocampal part of the cingulum bundle bilaterally. Resende et al. concluded that the integrity of white matter frontotemporal parietal fasciculi seems to play a role in the performance of episodic memory measured by the FMT among low-educated old-aged individuals.^57^

Two other studies from the same group investigated structural correlates of the performance on the FMT among different populations. In one study,^58^ 183 older adults (112 cognitively healthy, 26 with CIND and 45 with dementia), with median age of 78 years and median schooling of 4 years, were administered the FMT and submitted to 3-Tesla MRI to assess hippocampal volumes. The interaction between education and the left hippocampus, but not the right, predicted a significant variation in memory scores. The authors concluded that the left hippocampus seems to play a more important role in memory processing in more educated individuals.57 The second study^59^ compared brain atrophy patterns in MRI of patients with behavioral variant frontotemporal dementia (n=19) with and without episodic memory impairment defined by performance in the FMT of the BCSB (score <7 in the DR-FMT was used to classify into amnestic or non-amnestic bvFTD groups). Patients with bvFTD, AD dementia (n=21), and cognitively healthy controls (n=21) were matched by age, gender, and education. Compared with AD, amnestic frontotemporal dementia patients had more atrophy in the left fusiform cortex, left insula, left inferior temporal gyrus, and right temporal pole, whereas patients with AD had more atrophy in the left hippocampus, left frontal pole, and left angular gyrus.^59^

In a study to investigate the evolution of cognitive decline in patients with MCI, the BCSB was used at baseline and to assess evolution after 2 years of follow-up. The main finding of this study was that the group with a decline only in executive functions had more depressive symptoms and a lower conversion rate to dementia than the group with multiple decline.^60^

In a cross-sectional study, the association between the degree of adherence to Mediterranean and Mediterranean-DASH Intervention for Neurodegenerative Delay Diet (MIND) diet and cognitive performance was evaluated. The MMSE and BCSB were applied to classify 96 subjects into normal controls (NC), individuals with MCI, and AD. The conclusion was that moderate adherence to these diets may be associated with better cognition cognitively unimpaired individuals living in Brazil.^61^

In a study aimed to correlate functional capacity with cognition in a community-dwelling older-adult sample, the University of California, San Diego (UCSD) Performance-based Skills Assessment (UPSA) and cognitive screening tests, including the BCSB, were used. Significant correlations were found between scores in the UPSA and CDT and Verbal Fluency tests but not with the FMT scores.^62^

Cognitive deficits may be present in patients with mild traumatic brain injury (mTBI) in the first 24 hours after trauma (aTBI). For investigating the presence of cognitive deficits, the FMT of the BCSB, MMSE, Frontal Assessment Battery, and digit-span were used. Fifty-three patients with mTBI and 28 healthy controls participated in the study. Compared to controls, mTBI patients exhibited significantly worse performance on several tests (MMSE, Frontal Assessment Battery -FAB, and all tests of the FMT).^63^

Many patients with epilepsy complain of cognitive deficits, but usually are not submitted to neuropsychological evaluation due to several reasons, from lack of institutions in remote areas to low number of validated tests. A study used the BCSB to evaluate cognition of a sample of 371 individuals aged (18 years old with different types of epileptic syndromes, comparing the performance in the subtests with that of a control group with 95 individuals. The results showed some level of impairment in several domains, with lower performance on naming, memory (Immediate Memory, Learning, DR-FMT, Recognition of the FMT), CDT, phonemic Verbal Fluency, and the MMSE. There was a significant relationship between cognitive impairment and certain aspects of epilepsy (earlier onset, number of drugs taken), but not for the type of epileptic syndrome in this sample. The BCSB proved to be effective as a cognitive assessment screening test for epilepsy in adults.^64^

To investigate the impact of a sedentary life-style in a cross-sectional study, 120 individuals with diabetes type II and systemic arterial hypertension were examined in a cross-sectional study. The sample included 35% men and 65% women, with a mean of 64±9 years of age and who were evaluated by anthropometric variables, pedometer recordings, and BCSB. A high rate of physical inactivity was found among those enrolled in this project. Low level of daily physical activity was apparently correlated with cognitive dysfunction.^65^

Vitturi et al. evaluated the frequency and clinical predictors of cognitive impairment in ankylosing spondylitis (AS) using questionnaires and cognitive screening tests. The performance of patients with AS was lower than that of healthy controls in verbal fluency and CDT of the BCSB and in the Montreal Cognitive Assessment (MoCA).^66^

A recent study investigated the accuracy of the BCSB in the differential diagnosis between AD, non-AD cognitive impairment (both defined by cerebrospinal fluid AD biomarkers) and healthy cognition. The study included 117 individuals, being 45 with MCI or mild dementia within the AD continuum defined by the AT(N) classification (i.e., A+), 27 non-AD (A-) MCI or mild dementia patients, and 45 cognitively healthy individuals. DR-FMT showed high accuracy (AUROC=0.87) for discrimination between AD and non-AD versus controls. DR-FMT also displayed high accuracy (AUROC=0.91) for differentiation between AD and controls. Cerebrospinal fluid levels of the three AD biomarkers presented a significant, albeit weak, correlation with DR-FMT.^67^

These 37 studies published since 2008 found in the literature demonstrate that the BCSB, and particularly the DR-FMT, had an impact on the Brazilian research in clinical neurosciences, by allowing the use of tests with less impact of education.

At the end of 2020, Araujo et al.^68^ published the translation and diagnostic accuracy of the Brazilian version of the European Cross-Cultural Neuropsychological Test Battery (CNTB). This battery, which was designed for diagnosing dementia in immigrants or low-educated individuals living in European countries, consists of single tests.^69^ Among them, the test with the highest accuracy in differentiating patients with AD from cognitively healthy controls is the Recall of Pictures Test (RPT), particularly the delayed recall subtest.68,69 According to Araujo et al., this test is “similar to a test from the Brief Cognitive Screening Battery (BCSB), except that color pictures are used.”^68^

The figures in the FMT and the RPT are identical (except for the bucket, which was replaced by a trash can). The testing procedure is the same, and in both FMT and RPT, the semantic Verbal Fluency (animals in one minute) and the CDT are included as interference tests between Learning (encoding) and Delayed Recall. We wrote a letter to the Brazilian Journal of Psychiatry to demonstrated that the RPT of the CNTB is the color version of the FMT of the BCSB.^70^

We received a reply from the authors stating that they recognize that RPT was inspired by our work with the BCSB, but disagree that the RPT is simply a color version of the BCSB memory test.^71^

## DISCUSSION

The aim of the present review was to revisit the history of the BCSB, in particular the DR-FMT, and to document its trajectory as a clinical tool. In all, 59 studies using the battery were identified and briefly described. It is possible that a larger number of studies have included the BCSB. We are aware that the battery has been used in other papers and in the grey literature, but they are not easily reachable as the names of tests are often not included in the title or abstract.

It is possible to see how much this test has been used in Brazil and how it has allowed the inclusion of participants with low education or populations with heterogeneous educational level in several types of studies. The absence or very small impact of education was reported by many investigators, in diverse settings and regions.

The FMT has proven to be an easy and friendly test to be used in different scenarios, from epidemiologic to clinical studies. It takes three minutes longer than the MMSE, but the BCSB has more memory and executive functions tasks than the MMSE, and it does not need different cut-off scores according to education.

Notwithstanding, we are not aware of the use of the FMT of the BCSB in other Latin American countries or in other regions of the world where populations also have heterogeneous educational backgrounds.

Perhaps the BCSB and the FMT have not been used more frequently or in other countries because it was not so easily available and the cutoff scores for dementia diagnosis had not been clearly stated so far.

Now, with the definition of ≤5 as the best score for the diagnosis of dementia with the DR-FMT, it is more likely that the BCSB will be used more widely. Other important cutoff scores when using the BCSB are those for Verbal Fluency (animals in one minute). The cutoff obtained in two studies are ≤9 for illiterates, ≤12 for individuals with 1‒7 years of schooling, and ≤13 for those with 8 years or more of schooling.^72^ ^,^ ^73^

For the CDT, studies have shown that the best cutoff score ranged from ≤(4 in low educated individuals to ≤(9 in subjects with medium and high levels of education.^6^ ^,^ ^13^ These data show that the CDT is not a good test for diagnosis of dementia in populations with heterogeneous educational background, but although it is important for the interference phase of FMT.^19^

Regarding the debate on whether the RPT of the CNTB is the color version of the FMT of the BCSB, we believe that the most important fact is that this simple test, in its different versions, has demonstrated high sensitivity and specificity for the diagnosis of episodic memory impairment and dementia in different parts of the world and among people with different cultural background, such as individuals living in different regions of Brazil and immigrants or low-educated individuals living in European countries. This information should be disseminated to make this test even more widely known and used in several parts of the world where people with heterogeneous education live.

We want to thank the Brazilian researchers who included the FMT of the BCSB in their studies and helped to show its strength as a cognitive evaluation tool. Now, with this paper and the information on how to apply the BCSB, we hope that the investigation of cognitive decline is going to be easier in countries with heterogeneous educational backgrounds.
