# Erratum

**DOI:** 10.1590/1980-57642021dn15-020004erratum

**Published:** 2021-10-25

In the manuscript “The Figure Memory Test: diagnosis of memory impairment in populations with heterogeneous educational background”, DOI: 10.1590/1980-57642021dn15-020004 published in the Dement Neuropsychol. 2021;15(2):173-185.

**Where it reads:**

Sonia Maria Dozzi Bucki

**It should read:**

Sonia Maria Dozzi Brucki

